# Dysmorphic Features, Frontal Cerebral Cavernoma, and Hyperglycemia in a Girl with a De Novo Deletion of 7.23 Mb in Region 7p13-p12.1

**DOI:** 10.4274/jcrpe.4324

**Published:** 2017-12-15

**Authors:** Gilberto Pérez López, Beatriz Villafuerte Quispe, María José Cabrejas Núñez, Luis Castaño, Raquel Barrio

**Affiliations:** 1 Ramón y Cajal University Hospital, Department of Pediatrics, Pediatric Endocrinology and Diabetes Unit, Madrid, Spain; 2 Ramón y Cajal University Hospital, Department of Clinical Genetics, Madrid, Spain; 3 Cruces University Hospital, Research Unit, Bilbao, Spain

**Keywords:** Glucokinase, cerebral cavernous malformation, maturity-onset diabetes, oxoglutarate dehydrogenase, hyperglycemia, intellectual disability

## Abstract

We describe the case of a 7-year-old girl referred to our diabetes unit for hyperglycemia associated with facial dysmorphic features, intellectual disability, and cerebral cavernomas. Based on presence of anti islet antigen-2 (IA2) antibodies and a human leukocyte antigen of DR3/DR4/DQ2, the patient was initially diagnosed to be a case of type 1 diabetes mellitus. At follow-up, the very good metabolic control on a low insulin dose and negative IA2 antibodies led to a suspicion of glucokinase (GCK)-related maturity-onset diabetes of the young (MODY 2). This suspicion was substantiated in multiplex ligation-dependent probe amplification (MLPA) which showed a heterozygous GCK deletion (exons 1 to 12). However, the patient’s parents did not have such a deletion and were clinically euglycemic. Given the clinical picture and the MLPA findings, array based comparative genomic hybridization was performed showing a monoallelic deletion of 7.23 Mb in the short arm of chromosome 7 (7p13-p12.1). The deleted intervals contain 39 genes listed in the Online Mendelian Inheritance in Man list, including GCK associated with MODY 2, CCM2 associated with type 2 cerebral cavernous malformations, IGFBP-3 associated with decrease in postnatal growth, and OGD associated with alpha-ketoglutarate dehydrogenase deficiency, with cognitive impairment and movement abnormalities. This previously unreported deletion was considered to explain the clinical picture of the patient. Also, the findings suggest that 7p13-p12.1 contains genes involved in intellectual disability and craniofacial development.

**What is already known on this topic?**

Most glucokinase mutations are single nucleotide mutations. Glucokinase deletion mutations are very rare. Until now, a whole glucokinase deletion in non-syndromic patients has only been reported once. In syndromic patients, whole glucokinase gene deletions have been reported in combination with multiple gene deletions.

**What this study adds?**

Our patient with hyperglycemia and dysmorphic features had a deletion of 7.23 Mb comprising the region 7p13-p12.1, with involvement of 39 Online Mendelian Inheritance in Man genes, including glucokinase associated with maturity-onset diabetes of the young, CCM2 associated with type 2 cerebral cavernous malformations, insulin-like growth factors binding protein-3 associated with decreased postnatal growth, and oxoglutarate dehydrogenase associated with alpha-ketoglutarate dehydrogenase deficiency (short stature, hypotonia, cognitive impairment, and movement abnormalities). This previously unreported deletion explains the clinical picture of the patient and suggests that 7p13-p12.1 contains genes involved in intellectual disability and craniofacial development.

## INTRODUCTION

Hyperglycemia is a frequent cause for presentation at pediatric endocrinology clinics. Autoimmune diabetes and insulin resistance associated with obesity or with monogenic diabetes (mainly maturity-onset diabetes of the young) are among the major causes of hyperglycemia. In Spain, mutations in GCK and HNF-1α explain most cases clinically diagnosed maturity-onset diabetes of the young (MODY) (1). Pedigrees with monogenic MODY-type diabetes typically show a dominant pattern of inheritance.

We describe the case of a 7-year-old girl referred to our diabetes unit for hyperglycemia associated with dysmorphic features, intellectual disability, frontal cerebral cavernoma, and no familial antecedents of diabetes. In the genetic study, we identified a deletion of 7.23 Mb in the 7p13-p12.1 region (genomic coordinates Chr7: 42807167 to 50040279).

## CASE REPORT

A 7-year-old girl presented to our diabetes unit with hyperglycemia. In the previous 4 years, her fasting blood glucose levels were reported to vary between 103-135 mg/dL. There was no history of polyuria, polydipsia, or polyphagia. Likewise, there was no history of weight loss or of any episode of ketosis.

**Figure 1:**
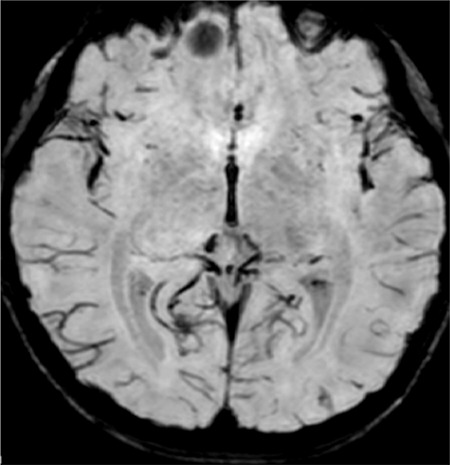
Cerebral magnetic resonance: Shows a frontal cerebral cavernoma (7x6 mm)

Due to detection of bradycardia, the patient was delivered by caesarean section at a gestational age of 40 weeks. Her Apgar score was 6/9 and type 3 reanimation was needed. Birth weight was 2530 g [-2 standard deviation (SD)], birth length 47 cm (-1.7 SD), and head circumference was 35 cm (0.5 SD). No hypoglycemia or hyperbilirubinemia were noted in her neonatal life. However, she received early stimulation, starting at age 22 months, for language delay and hypotonia. She was reported to have three febrile seizure episodes and also tics. She showed poor school performance and poor social interaction. For these reasons, she was referred to the pediatric neurology clinic where she was diagnosed to have a frontal-cerebral cavernoma accompanied by intellectual disability and dysmorphic features (Figure 1).

**Family history:** She had healthy non-consanguineous parents [mother’s height and father’s height were 162 cm (0.13 SD) and 165.5 cm (-1.6 SD), respectively] and a healthy sister. There was no family history of diabetes, neurological or hereditary metabolic disorders.

**Figure 2:**
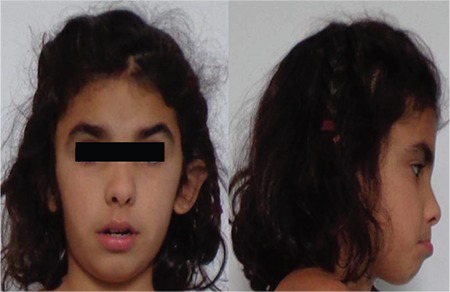
Facial dysmorphic features: Triangular face, short front, depressed nasal bridge, low hair implantation, bushy eyebrows, synophridia, and microretrognathia

**Physical examination:** Her weight and height were 20.5 kg (-0.96 SD) and 115.4 cm (-1.6 SD), respectively, with a body mass index of 15.4 kg/m2 (-0.4 SD). Her target height was 157.3 cm (-1.1 SD). She had dysmorphic features with a triangular face, short forehead, depressed nasal bridge, low hair implantation, bushy eyebrows, synophridia, and microretrognathia (Figure 2). The rest of the examination was normal.

**Initial genetic studies:** In the initial study of the dysmorphic features and mental retardation, we determined the karyotype (46XX), investigated the subtelomeric regions and the FMR1 gene, which were normal. Other laboratory tests were also ordered.

**Biochemical analyses:** Hemoglobin A1c (HbA1c) level was 6.0%. Oral glucose tolerance test (OGTT) (with 1.75 g/kg) revealed a basal glycemia of 129 mg/dL, a serum glucose level of 191 mg/dL after 1 hour and 150 mg/dL after 2 hours, a sign of impaired glucose tolerance. She had preserved her pancreatic reserves. In the course of the OGTT, c-peptide levels at 0, 60, 120 minutes were 0.8, 3.7 and 2.1 ng/mL, respectively. Thyroid, liver and kidney functions were normal.

**Autoimmunity:** The patient’s anti-IA2 (anti-tyrosine phosphatase) level was 78.8 U/mL (positive >7.5 U/ mL), and she did not have any anti-glutamic acid decarboxylase (GAD-0.0 U/mL). Human leukocyte antigen (HLA) determination revealed HLA-DR3/DR4 and HLA-DQ2.

**Diagnosis:** Given the presence of fasting hyperglycemia (129 mg/dL), the positivity for a pancreatic autoantibody (anti-IA2), and a HLA trait with increased risk for development of autoimmune diabetes (HLA DR3/DR4), a diagnosis of type 1 diabetes mellitus (T1DM) was made and treatment with insulin at 0.3 U/kg/day was started.

**Follow-up:** Nine months after T1DM diagnosis, the patient presented with a very good metabolic control while on a low insulin dose (0.15 U/kg/day). Her weight was 20.8 kg (-1.9 SD) and her height was 118.9 cm (-1.7 SD), with a linear growth velocity (LGV) of 4.38 cm/year (-1.5 SD). Her laboratory results were negative for anti-IA2 and anti-GAD antibodies (samples analyzed in two different laboratories). HbA1c was 5.8%. Insulin-like growth factors (IGF)-1 and IGF binding protein-3 (IGFBP-3) levels, adjusted for age and sex, were 104.2 ng/mL (-0.6 SD) and 2947 ng/mL (-1 SD). The above findings suggested the need to further investigate an alternative diagnosis for her hyperglycemia.

GCK-related MODY 2 was suspected by persistent mild hyperglycemia in the fasting state. We requested a multiplex ligation-dependent probe amplification (MLPA) that showed a heterozygous GCK deletion (exons 1 to 12). However, the patient’s parents did not have such a deletion and were clinically euglycemic. Previously, we had performed karyotype analysis to the parents of the patient and both were found to be normal, discarding chromosome translocations. These results increased the likelihood that the patient’s deletion would be de novo.

**Figure 3:**
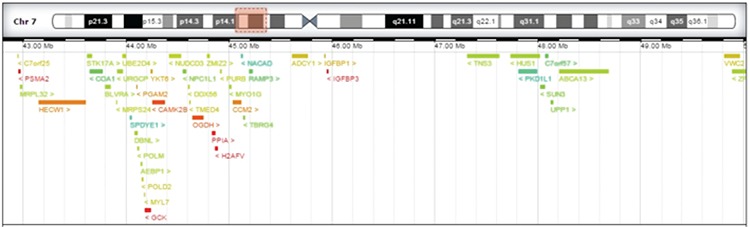
Region 7p13-p12.1 (genomic coordinates Chr7: 42807167 to 50040279): copy number variation 7.23 Mb contains 39 genes included in Online Mendelian Inheritance in Man list

**Results of Array Based Comparative Genomic Hybridization (International System for Human Cytogenetic Nomenclature 2013):** Given the clinical picture and the MLPA findings, an array based comparative genomic hybridization (CGH) (KaryoNIM® Postnatal 60k) was performed. This array-CGH platform is primarily used for intellectual disabilities and syndromes with multiple malformations. This method simultaneously detects the presence or absence of genetic and chromosomal variations (duplications or deletions) responsible for 160 genetic syndromes with a minimum resolution of approximately 275 kilobases between probes. The results revealed a pattern of arr[hg19] 7p13p12.1(42,807,167-50,040,279)x1 (female genomic pattern). This result shows a deletion of 7.23Mb in the 7p13-p12.1 region (genomic coordinates Chr7: 42807167 to 50040279). This region (Figure 3) contains 39 genes included in Online Mendelian Inheritance in Man (OMIM) (2) database. GCK associated with MODY 2, CCM2 associated with type 2 cerebral cavernous malformations, OGDH associated with alpha-ketoglutarate dehydrogenase deficiency (short stature, cognitive impairment, and movement abnormalities), and IGFBP-3 whose deletion has been associated with a 20% decrease in postnatal growth are among these genes (3). Deletion of these genes may explain patient’s clinical signs. With these findings, the decision was taken to discontinue insulin therapy.

At 8 years and 10 months old, the patient continued to have good metabolic control (HbA1c <6.0%) without insulin, although with a slowing in growth [height 122.2 cm (-1.9 SD) with a LGV of 3.5 cm/year (-2.3 SD)]. For this reason, IGFBP-3 deficit is being reevaluated again.

## DISCUSSION

Our patient had hyperglycemia with positive anti-IA2 antibodies. These findings and presence of HLA findings associated with risk for development of autoimmune diabetes (HLA DR3/DR4) were initially interpreted as signs of T1DM. At follow-up, and after nine months of insulin treatment, she presented in a state of very good metabolic control on a low insulin dose, with anti-IA2 antibodies which had become negative. These findings led us to further investigate for presence of other underlying causes of hyperglycemia.

Monogenic diabetes MODY 2 was suspected (mutations in GCK explains most cases of monogenic causes of diabetes in Spain) and MLPA was performed in the patient and her parents, showing an heterozygous GCK gene deletion and wild type results, respectively.

The presence of dysmorphic features and of a frontal cavernoma increased the suspicion of a genetic syndrome. Given the clinical picture and the finding in MLPA, array-CGH (KaryoNIM® Postnatal 60k) was performed, finding the real size of the deletion being 7.23 Mb long in the region 7p13-p12.1.

According to the “Database of Chromosomal Imbalance and Phenotype in Humans Using Ensembl Resources” (DECIPHER) (4), the 7p13-p12.1 deleted interval contains five genes (GCK, IGFBP-3, OGDH, PPIA, and PSMA2) associated with low haploinsufficiency (HI) score (HI index <10%) (5) and/or high loss intolerance score (pLI ≥0.9) (6) and one intermediate (CMM2: HI 28.18% and pLI 0.48). Four of these genes described (Table 1) are consistently linked to clinical features observed in our patient, namely, mild hyperglycemia in the fasting state, cerebral cavernous malformations, decrease in postnatal growth, cognitive impairment, movement disorders (tics), and hypotonia. The clinical impact of deletion in this region is unknown, so close clinical monitoring is needed.

Most GCK mutations are single nucleotide mutations (7). GCK deletion mutations are very rare (8). Until now, a whole GCK deletion in non-syndromic patients have only been reported once (9). In syndromic patients, whole GCK gene deletions have been reported in combination with multiple gene deletions (10). The low birth weight (-2 SD) in this patient can be explained by the mother not having the GCK mutation and the fetus having the GCK mutation, which indirectly decreases fetal insulin secretion and thereby fetal growth (11,12).

At follow-up, the patient was found to have an altered postnatal growth (height -1.9 SD and LGV -2.3 SD) that could be explained in part by the HI of IGFBP-3 and OGHD. IGFBP-3 possesses both growth-inhibitory and potentiating effects on cells that are independent of IGF action and are mediated through specific IGFBP-3-binding proteins/receptors located at the cell membrane, cytosol, or nuclear compartments and in the extracellular matrix. IGFBP-3 deletion has been associated with a 20% decrease in postnatal growth (3).

The patient received early stimulation, starting at age 22 months, for hypotonia and language delay. OGDH HI is associated, according to the Human Phenotype Ontology (13), with short stature, hypotonia, cognitive impairment, and movement abnormalities, among others features. For this reason, the patient was referred to the hereditary metabolic diseases unit for further assessment.

After a search in DECIPHER, we have not found another case with the same deletion. But we found a boy (DECIPHER ID 277032) with a more extensive deletion (9.26 Mb genomic coordinates Chr 7: 44085112-53341792) that includes the region 7p13-p12.1. This copy number variation contains 36 genes included in OMIM list. PPIA, IGFBP-3, GCK, GRB10, and H2AFV have HI <10% and that patient’s phenotype includes global developmental delay, tall stature, macrocephaly, depressed nasal bridge, pectus excavatum, and hypospadias. Surprisingly, the patient had euglycemia and tall stature.

To summarize, our patient with hyperglycemia and dysmorphic features had a deletion of 7.23 Mb comprising the region 7p13-p12.1, with involvement of 39 OMIM genes, including: GCK associated with MODY 2, CCM2 associated with type 2 cerebral cavernous malformations, IGFBP-3 associated with decreased postnatal growth, and OGDH associated with alpha-ketoglutarate dehydrogenase deficiency. This previously unreported deletion is thought to explain the clinical picture of the patient and suggests that 7p13-p12.1 contains genes involved in intellectual disability and craniofacial development.

## Figures and Tables

**Table 1:**
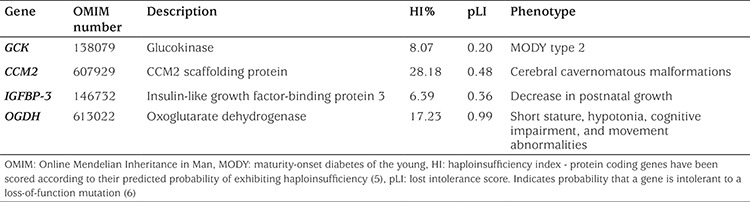
Genes in deleted region (7p13-p12.1) and predicted phenotypic effects
